# Natural and Vaccine-Mediated Immunity to *Salmonella* Typhimurium is Impaired by the Helminth *Nippostrongylus brasiliensis*

**DOI:** 10.1371/journal.pntd.0003341

**Published:** 2014-12-04

**Authors:** Saeeda Bobat, Matthew Darby, Dunja Mrdjen, Charlotte Cook, Erin Logan, Jennifer Auret, Elizabeth Jones, Corinna Schnoeller, Adriana Flores-Langarica, Ewan A. Ross, Alykhan Vira, Constantino López-Macías, Ian R. Henderson, James Alexander, Frank Brombacher, William G. Horsnell, Adam F. Cunningham

**Affiliations:** 1 MRC Centre for Immune Regulation, Institute of Microbiology and Infection, School of Immunity and Infection, College of Medical and Dental Sciences, University of Birmingham, Birmingham, United Kingdom; 2 Institute of Infectious Disease and Molecular Medicine, Faculty of Health Sciences, University of Cape Town, Observatory, Cape Town, South Africa; 3 Department of Life Sciences, South Kensington Campus, Imperial College London, London, United Kingdom; 4 Medical Research Unit on Immunochemistry, Specialties Hospital, National Medical Centre “Siglo XXI” Mexican Institute for Social Security (IMSS), Mexico City, Mexico; 5 Strathclyde Institute of Pharmacy and Biomedical Sciences, University of Strathclyde, Glasgow, United Kingdom; Universidad San Francisco de Quito, Ecuador

## Abstract

**Background:**

The impact of exposure to multiple pathogens concurrently or consecutively on immune function is unclear. Here, immune responses induced by combinations of the bacterium *Salmonella* Typhimurium (STm) and the helminth *Nippostrongylus brasiliensis* (Nb), which causes a murine hookworm infection and an experimental porin protein vaccine against STm, were examined.

**Methodology/Principal Findings:**

Mice infected with both STm and Nb induced similar numbers of Th1 and Th2 lymphocytes compared with singly infected mice, as determined by flow cytometry, although lower levels of secreted Th2, but not Th1 cytokines were detected by ELISA after re-stimulation of splenocytes. Furthermore, the density of FoxP3+ T cells in the T zone of co-infected mice was lower compared to mice that only received Nb, but was greater than those that received STm. This reflected the intermediate levels of IL-10 detected from splenocytes. Co-infection compromised clearance of both pathogens, with worms still detectable in mice weeks after they were cleared in the control group. Despite altered control of bacterial and helminth colonization in co-infected mice, robust extrafollicular Th1 and Th2-reflecting immunoglobulin-switching profiles were detected, with IgG2a, IgG1 and IgE plasma cells all detected in parallel. Whilst extrafollicular antibody responses were maintained in the first weeks after co-infection, the GC response was less than that in mice infected with Nb only. Nb infection resulted in some abrogation of the longer-term development of anti-STm IgG responses. This suggested that prior Nb infection may modulate the induction of protective antibody responses to vaccination. To assess this we immunized mice with porins, which confer protection in an antibody-dependent manner, before challenging with STm. Mice that had resolved a Nb infection prior to immunization induced less anti-porin IgG and had compromised protection against infection.

**Conclusion:**

These findings demonstrate that co-infection can radically alter the development of protective immunity during natural infection and in response to immunization.

## Author Summary

Vaccination studies in animal models have focused on understanding responses in young, previously naïve mice. In reality, humans are vaccinated or respond to infection in the context of a life-time of accumulated exposure to multiple, systemic infections and other vaccines, some of which are themselves attenuated live organisms. This is even more pronounced in areas that are endemic for infectious diseases. We wished to examine the impact infectious history can have on the immune response against infection and the efficacy of vaccination. To do this, we used two classes of pathogens that model clinically important invasive infections. One pathogen is the bacterium, *Salmonella* Typhimurium against which we have also developed an experimental porin vaccine, and the second is an invasive helminth, *Nippostrongylus brasiliensis*, that models aspects of hookworm infections. Our studies indicate that exposure to a second, unrelated pathogen can reduce the efficiency of immunity generated during natural infection and immunity generated after vaccination. These results are important as they help to identify potential strategies for improving immune-mediated control of infection and the success of vaccination in infection-endemic regions.

## Introduction

Models examining immunity to experimental infections primarily focus on responses to a single pathogen or vaccine in an immunologically naïve host. Such studies have shaped our understanding of how infections develop and are controlled. However, in reality individuals are exposed to multiple pathogens, often concurrently during their life-time [1]–[4]. Whether infectious history may influence the type of immune response mounted by the host to a new vaccine pathogen has not been extensively explored.

Of particular significance is that regions endemic for non-typhoidal *Salmonella* (NTS) serovars, such as *Salmonella* Typhimurium (STm) [5] are also endemic for parasitic nematode infections, such as hookworm [6]. This provides opportunities for concomitant STm and helminth infections to develop. In distinct forms both infections can be modelled in a murine system. *Nippostrongylus brasiliensis* (Nb), a natural parasite of rats is used as a model infection in mice of human hookworm disease. Nb induces Th2 features such as interleukin 4 (IL-4), IL-13, IgG1 and IgE [7]–[14]. Infection with Nb in mice is self-limiting, with worms cleared from BALB/c mice in a narrow period of 9–11 days post-infection when mice are infected with the common dose of 500–750 L3 larvae [7], [11]. Having these defined kinetics for clearance enables identification of factors that interfere with immunity. Exposure to an additional agent after resolution of Nb infection enables any lasting influence of helminth infection on host immunity to the second antigen to be identified.

Clearance of STm infections require Th1-mediated immunity, characterized by the induction of Interferon (IFN) γ and IgG2a in mice [15]–[18]. A mutation in the Slc11a1/Nramp gene renders mouse strains, such as BALB/c, hyper-susceptible to virulent strains of STm whilst attenuated strains are cleared gradually. For the latter strains, such as the AroA-deficient STm strain SL3261, clearance is achieved 1–2 months after infection with a typical dose of 5×10^5^ bacteria administered systemically [19]–[21]. A striking component of host immunity to attenuated STm is a rapid and extensive extrafollicular (EF) antibody response with switching to IgG2a and IgG2b, which occurs without parallel germinal centre (GC) induction [19].

While B cells and antibody are wholly dispensable for controlling primary STm murine infections [20], [22], [23], the presence of antibody to STm prior to infection can be protective [19], [22], [24], [25]. Indeed, we have found that immunization with purified porins induced antibody sufficient to protect against subsequent STm infection [26], with IgG augmenting the protection afforded by IgM. Thus, factors that influence IgG responses are likely to affect protection and immunization with porin proteins.

Helminth infections may modulate responses to other pathogens [27]–[29] and to vaccination [30]–[33], although the nature of these influences have not been fully elucidated. Furthermore, such studies have often not addressed the impact of co-infection on the immunological response to each infection. In this study, we investigated the development and efficacy of immune responses after immunization with combinations of Nb, STm and porins. Our data shows that co-infection with Nb and STm impairs clearance of both pathogens. Whilst some changes in cytokine patterns were observed, the pathogen-associated pattern of isotype-switching was conserved so that specific IgG1, IgE and IgG2a responses all developed in parallel. Furthermore, prior Nb infection impaired the protective efficacy of porin immunization indicating a longer-term impact of helminth infection, suggesting these effects were not necessarily dependent upon an active infection. These data not only further our understanding of the relationship between host and pathogen and the mechanisms used to regulate immune function, but also identify the need to consider the impact of infectious history on the host's capacity to implement protective immunity.

## Methods

### Mice used and ethical statement

Specific pathogen-free 6–8 week BALB/c mice were obtained from the animal facility at the University of Cape Town, South Africa. All animal procedures were carried out under Protocol 012-006 which was approved by the Animal Research Ethics Committee at the University of Cape Town. All procedures were also conducted in strict accordance with the South African code of practice for laboratory animal procedures.

### Immunogens

STm SL3261 is an attenuated strain of STm SL1344 [34]. Nb was maintained through passage in rats. Outer membrane preparations from STm were generated by 2% (vol/vol) Triton X-100 extraction [19]. Purified porins from STm (strain ATCC 14028) were generated as described previously [26], [35] using SDS and FPLC and suspended in PBS in 0.1% (wt/vol) SDS. Nb total antigen preparations were generated by snap-freezing L3 stage larvae and homogenizing by sonication. Antigen was stored until use at −80°C.

### Immunizations, infections and opsonisation of bacteria

Mice were infected intraperitoneally (i.p.) with 5×10^5^ STm SL3261. Tissue bacterial burdens were determined by direct culturing. Mice were infected subcutaneously (s.c.) with 500 Nb L3 larvae. Adult worm burdens were determined by counting in the gut lumen under a dissecting microscope as previously described [9]. Where stated, mice were immunized i.p. with 20 µg porins in PBS. Opsonizing bacteria with antisera was performed as described previously [19], [26]. A single serum was used per mouse and sera were heat-inactivated at 56°C for 0.5 h to inactivate complement. Bacteria (2.5×10^6^/mL) and sera (1∶100) were mixed for 1 h before infection. Bacterial viability and lack of agglutination were confirmed by plating.

### FACS

Splenic single cell suspensions were prepared and red cells were lysed with ACK lysing buffer (Gibco Life Technologies). Cells were initially blocked prior to staining with anti-CD16/32 antibody before surface staining for 20 min at 4°C with CD3-FITC (Clone 145-2C11), CD4-PerCP Cy 5.5 (Clone RM4-5). Intracellular cytokine staining was performed by ex-vivo re-stimulation as described previously [15]. Briefly, 5×10^6^ splenocytes were plated with 1 µg/ml anti-CD28 (clone 37.51) and re-stimulated in a pre-coated well with anti-CD3 (10 µg/ml) (clone 145-2C11). Cells were incubated for 2.5 h followed by 2.5 h with GolgiStop (BD Biosciences). Cells were then surface stained, fixed and permeabilised with Cytofix/Cytoperm Plus for 20 min at 4°C before intracellular cytokine staining using IL-13 PE (Clone JES10-5A2) or IFNγ-APC (Clone XMG1.2) or isotype controls (all BD Biosciences). Cells were acquired using a FACSCalibur (BD Biosciences) and analysed using FlowJo Software.

### Immunohistology

Immunohistology was performed on frozen sections as described previously [19], [36] with tissues frozen in liquid nitrogen. CD3, IgG2a, IgG1 (Clone LO-MG1-2), IgE (Clone LO-ME-2) and FoxP3 cells were detected using rat anti-mouse antibodies in conjunction with biotinylated rabbit anti-rat immunoglobulins. Signal was developed using streptavidin ABComplex alkaline phosphatase (DakoCytomation) with naphthol AS-MX phosphate with Fast Blue salt and levamisole. Sheep anti-mouse IgD binding was detected using horseradish peroxidase (HRP)-conjugated donkey anti-sheep immunoglobulins with Diaminobenzidine (Sigma Aldrich). Hamster anti-mouse CD3 binding was detected using goat anti-hamster IgG followed by HRP-conjugated donkey anti-sheep immunoglobulins with Diaminobenzidine. The area of the spleen occupied by germinal centres and cells per square millimeter were calculated using a point-counting technique as described previously [37].

### Serology

Enzyme-linked immunosorbent assay (ELISA) was performed as described previously [19]. NUNC Maxisorp plates were coated overnight with antigen at 5 µg/ml in coating buffer. Plates were then blocked with 1% BSA before serum was added in PBS-0.05% Tween-20 and diluted stepwise. Bound antibodies were detected using alkaline-phosphatase conjugated, goat anti-mouse secondary antibodies (Southern Biotech) and Sigma-Fast p-nitrophenylphosphate (Sigma Aldrich). The absorbance at ODλ_405_ nm was determined using an Emax microplate spectrophotometer (Molecular Devices, Germany). Relative reciprocal titres were calculated by measuring the dilution at which the serum reached a defined ODλ_405_ nm.

### Cytokine ELISA

Splenocytes (2×10^5^) were plated for 48–72 h with 1 µg/ml anti-CD28 (Clone 37.51) and re-stimulated with either 10 µg/ml anti-CD3 (Clone 145-2C11) which was pre-coated overnight or 10 µg/ml heat-killed STm. Heat-killed STm was prepared by heat inactivation at 72°C for 1 hour. Control wells were stimulated with anti-CD28 and PBS. Cytokines secreted into the supernatants were then measured using the appropriate ELISA Ready-Set-GO kit (eBiosciences) as per manufacturers' instructions. Briefly, plates were coated overnight with capture antibody, blocked for 1 h at room temperature with 2% fat-free milk in PBS, after which samples and standards were added overnight at 4°C. Biotinylated secondary antibodies were then added and signal detected using streptavidin-HRP and 3,3′,5,5′-tetramethylbenzidine solution before stopping with 1 M H_3_PO_4_. The absorbance at ODλ_450_ nm (background at ODλ_540_ nm) was determined using a Versamax tunable microplate spectrophotometer (Molecular Devices, Germany).

### Statistics

The data is expressed as the mean plus one standard deviation. Significant differences were determined using the Mann-Whitney non-parametric two-tailed test using GraphPad Prism Version 5. P≤0.05 was accepted as significant.

## Results

### Co-infection with Nb and STm impairs control of each pathogen

**Figure 1:**
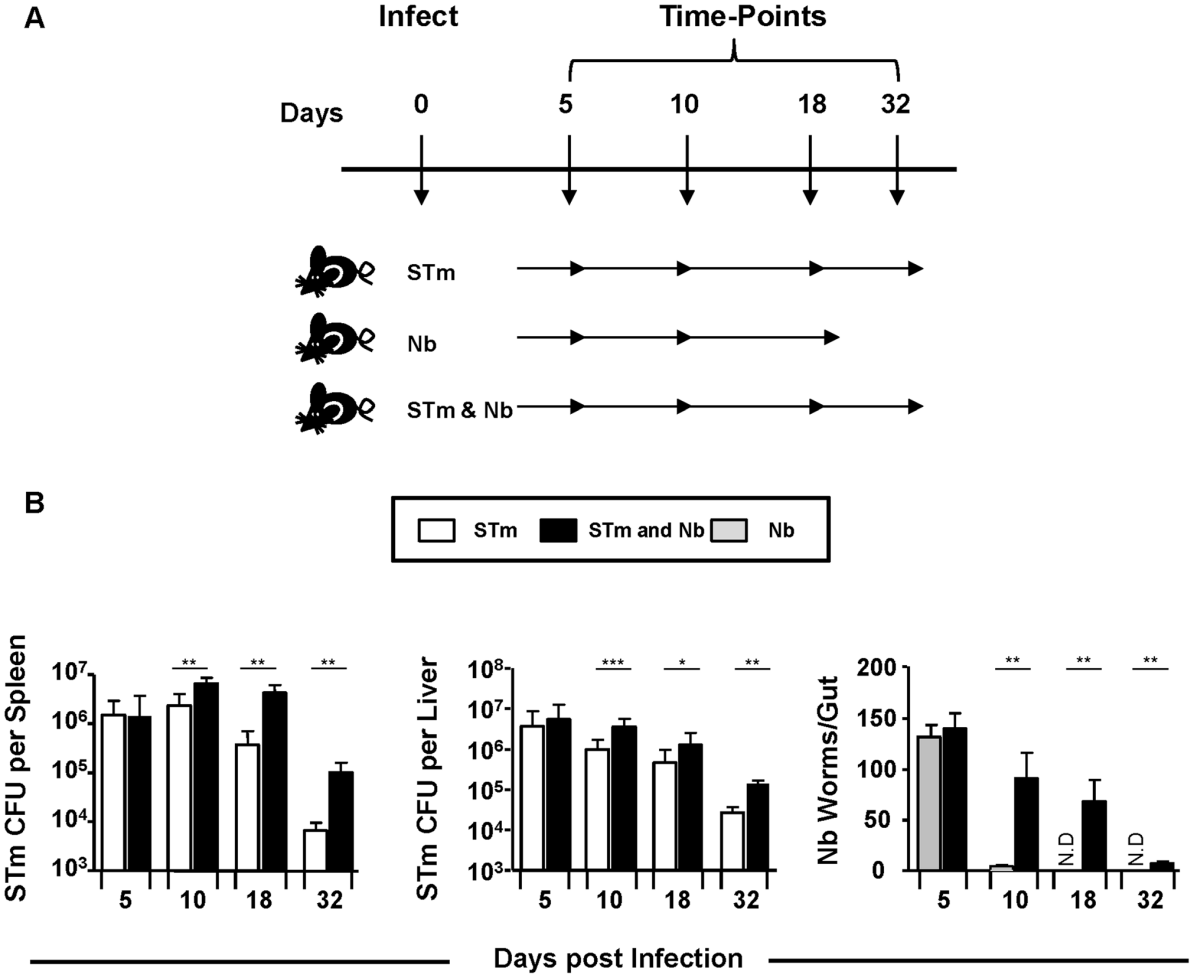
Co-infection with Nb and STm impairs control of each pathogen. **A**) WT mice were infected with either 5×10^5^ STm, 500 L3 Nb larvae or both for 5, 10, 18 or 32 days. **B**) Splenic and liver bacterial numbers were quantified from STm-infected animals. Small intestines were isolated and total worm burdens were assessed from Nb-infected mice. Infections with STm and Nb were administered intraperitoneally and subcutaneously respectively. Groups contained 4–6 mice with experiments performed twice for each time point. (N.D = Not detected, ^*^P<0.05, ^**^P<0.005 and ^***^P<0.0005).

To assess whether synchronous administration of STm and Nb altered the kinetics of clearance, we infected WT mice with either 5×10^5^ attenuated STm i.p., 500 L3 Nb larvae s.c., or both pathogens for 5, 10, 18 or 32 days (Figure 1A). While equivalent bacterial numbers were found in the spleens and livers of STm and co-infected mice at day 5 post-infection, after this time bacterial numbers were consistently higher in co-infected mice (Figure 1B). As expected intestinal worm burdens in Nb-only infected mice were largely cleared by day 10. However, co-infected mice demonstrated persisting Nb infection up to 32 days. Thus, co-infection with STm and Nb impairs immunity to both pathogens.

### Co-infection alters type-specific T helper responses to Nb but not STm

**Figure 2:**
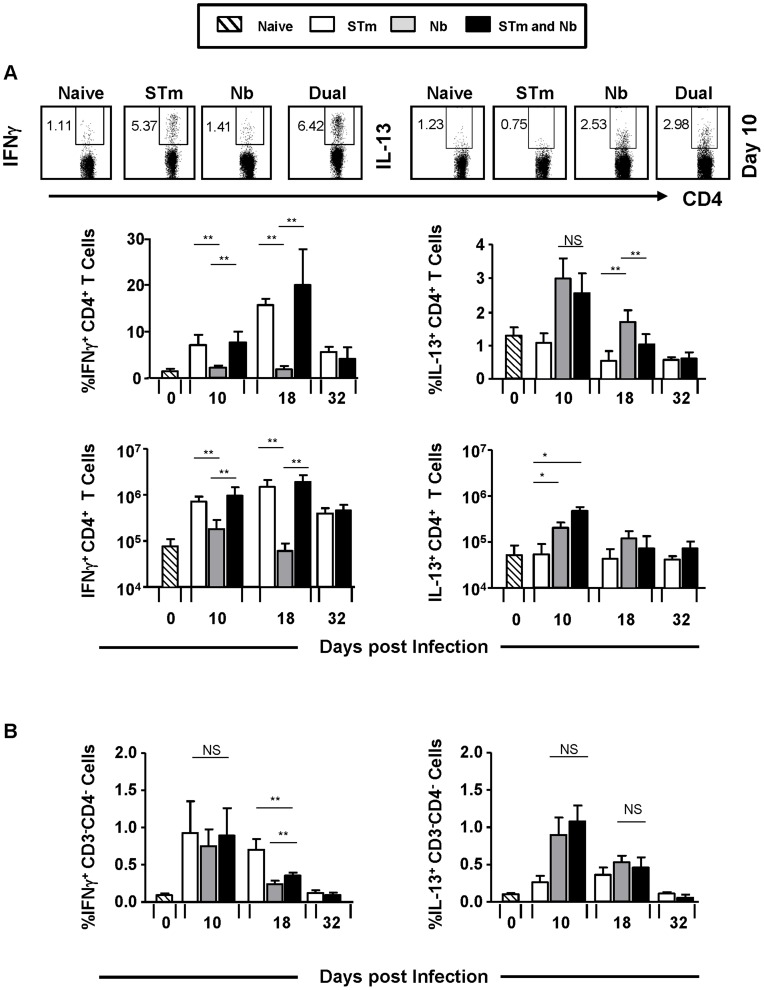
Co-infection does not prevent Th1 and Th2 cell polarization. Splenocytes from mice which were infected as in Figure 1A were re-stimulated ex-vivo with anti-CD3 in the presence of anti-CD28: IFNγ and IL-13 induction in **A**) CD3^+^CD4^+^ T cells and **B**) CD3^−^CD4^−^ cells was measured 6 hours post-stimulation by intracellular FACS and is represented as a proportion and/or absolute numbers. Infections with STm and Nb were administered intraperitoneally and subcutaneously respectively. Data is representative of 4–6 mice per group with experiments performed twice for each time point. (NS = Non-significant, ^*^P<0.05 and ^**^P<0.005).

**Figure 3:**
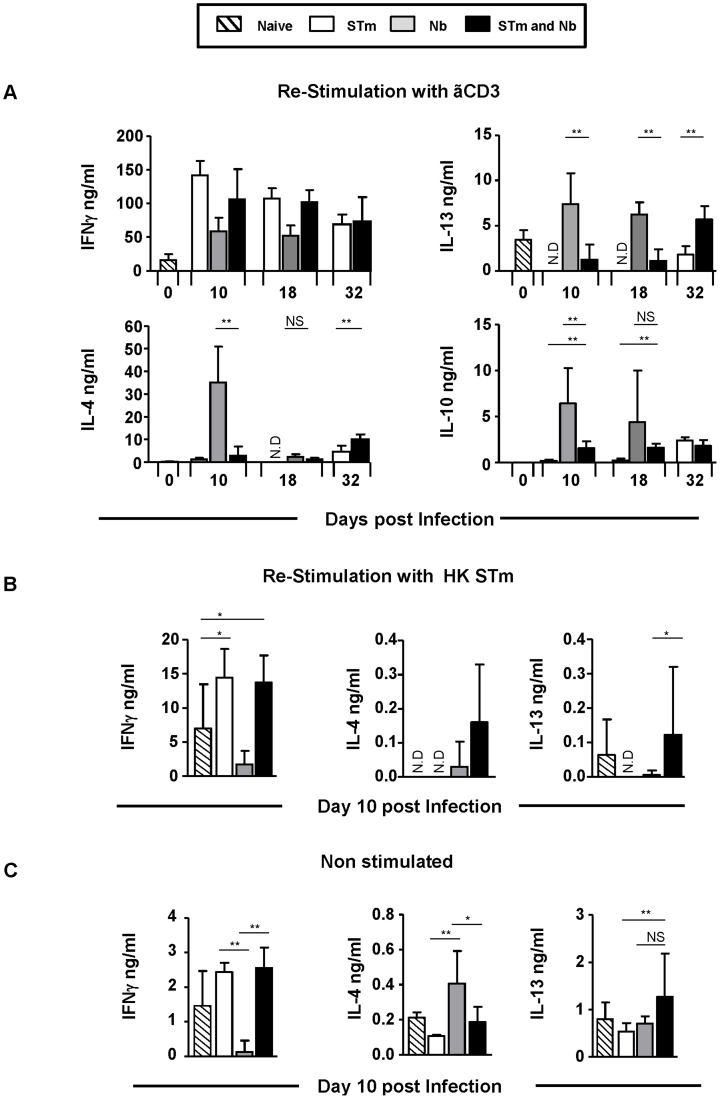
Co-infection alters type-specific T-helper responses to Nb but not STm. Splenocytes from mice which were infected as in Figure 1A were plated with anti-CD28 and re-stimulated ex-vivo with: **A**) anti-CD3 or **B**) 10 µg/ml heat-killed STm (HK_STm) and **C**) control wells were re-stimulated with PBS. IFNγ, IL-13, IL-4 and IL-10 secretion was measured by ELISA from supernatants 48–72 hours post-stimulation. Infections with STm and Nb were administered intraperitoneally and subcutaneously respectively. Data is representative of 4–6 mice per group with experiments performed twice for each time point. (NS = Non-significant, N.D = Not detected, ^*^P<0.05 and ^**^P<0.005).

The impact of co-infection on pathogen clearance suggested perturbed type-specific immunity to each pathogen. The proportion and numbers of T cells from co-infected mice that produced IFNγ or IL-13 after anti-CD3 re-stimulation were largely similar to that seen after single STm or Nb infection respectively, at all time-points (Figure 2A). As the capacity to induce pathogen-associated Th1 and Th2 cytokines was maintained, levels of secreted cytokines from splenocyte cultures were examined (Figure 3A). After re-stimulation with anti-CD3 in the presence of anti-CD28 secreted levels of IFNγ were similar between STm-only and co-infected mice at all time-points examined, reflecting the intracellular cytokine staining. In contrast, levels of IL-4 and IL-13 were greatly reduced at times after co-infection compared with Nb-only infected mice (Figure 3A). To examine if this reflected cytokine responses induced after re-stimulation with STm, splenocytes from day 10 infected mice were re-stimulated with heat-killed STm instead of anti-CD3 (Figure 3B). Levels of IFNγ were similar in both STm-infected groups but there was an increase in IL-4 and IL-13 in the co-immunized group. Therefore, co-infection has little impact on the development of Th1 and Th2 cytokine-producing T cells but can modulate the levels of cytokines secreted.

Cytokines from non-T cells can influence the functional response of T cells [38]. Therefore IFNγ or IL-13 expression in CD3^−ve^ cells was assessed by flow cytometry (Figure 2B). This showed that at all times after infection <1% of cells were positive for IFNγ or IL-13. Furthermore, co-infection did not dramatically alter the cytokine pattern seen after single infection. In addition, we examined cytokine secretion by splenocytes from mice infected for 10 days which were cultured without stimulation (Figure 3C). Cytokines were detected at lower levels than after anti-CD3 stimulation. IFNγ levels were similar in all groups except for the group that only received Nb, where they were lower. IL-4, but not IL-13, levels were reduced in co-infected mice compared to Nb only infected mice.

### Co-infection alters the frequency of T zone-localized FoxP3 cells

**Figure 4:**
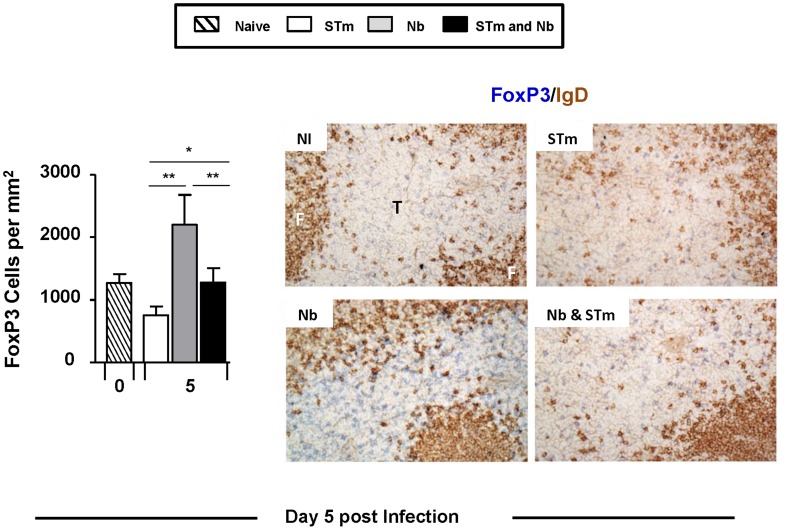
Co-infection alters the frequency of T-zone localised FoxP3 cells. Spleen sections were generated for immunohistology at day 5 post-infection from mice which were infected as in Figure 1A. Sections were double-stained for FoxP3 with CD3. These sections were then used to quantify FoxP3+CD3+ T cells in the T-zone per mm^2^. Representative images show double-staining with FoxP3 (blue) and IgD (brown) with images acquired using a Leica microscope DM6000 using a 20× objective. Infections with STm and Nb were administered intraperitoneally and subcutaneously respectively. Data is representative of 4–6 mice per group with experiments performed twice for each time point. T = T zone; F = B cell follicle (^*^P<0.05 and ^**^P<0.005).

Helminth infections are associated with the induction of T regulatory (Treg) cells [39]–[41]. Therefore, it is possible that co-infection could either augment or diminish Treg responses and the levels of associated IL-10 observed compared to each pathogen alone. Initially, levels of secreted IL-10 after re-stimulation with anti-CD3 were assessed by cytokine ELISA (Figure 3A). This revealed that IL-10 was readily detected after Nb infection, but after STm infection the levels were similar to those of non-infected cultures. When IL-10 was examined after co-infection it was found to be intermediate between the STm-only and Nb-only infected mice on days 10 and 18 (Figure 3A). Thus, the presence of STm was associated with a moderation in the levels of IL-10 detected in Nb-infected mice. Since Treg are significant sources of T cell-derived IL-10, the impact of co-infection on Tregs was assessed. To do this we used immunohistochemistry to examine the frequency of FoxP3+ T cells in the T zones of infected mice on day 5 after infection, when pathogen burdens were similar in mice that received one or both pathogens (Figure 1A). Reflecting the IL-10 results, the density of FoxP3+ cells in the T zones of co-infected mice was significantly lower relative to mice that were only infected with Nb, yet significantly higher than mice only infected with STm (Figure 4).

### Pathogen-induced patterns of immunoglobulin switching are maintained during co-infection

**Figure 5:**
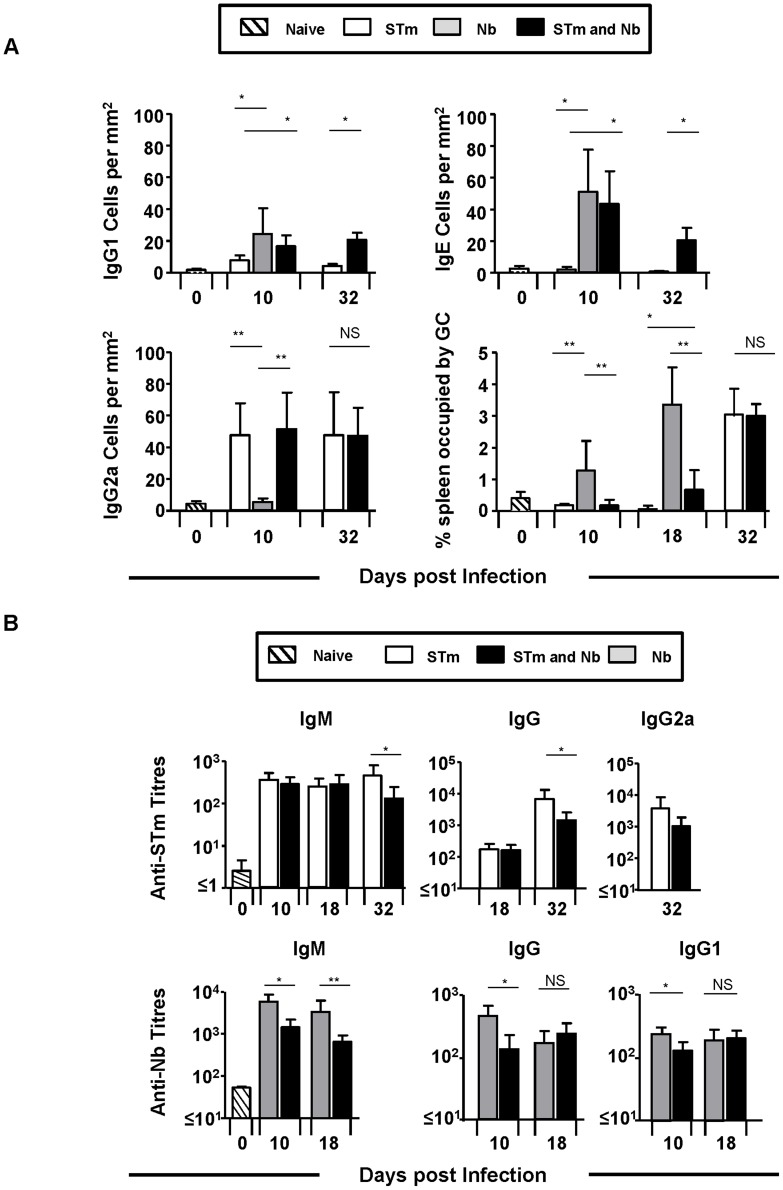
Immunoglobulin-switching patterns are maintained during co-infection but serum immunoglobulin responses to STm are reduced. **A**) Spleen sections were generated for immunohistology from mice which were infected as in Figure 1A. Sections were double-stained for IgD with IgG1, IgG2a, IgE or CD3. These sections were used to quantify extrafollicular plasma cells per mm^2^ and determine the proportion of the spleen occupied by germinal centres. Germinal centres were identified as areas of the follicle which were IgD^lo^. **B**) Serum anti-STm IgM, IgG, IgG2a and anti-Nb IgM, IgG and IgG1 antibody titres were quantified by ELISA against a total outer membrane preparation from STm and homogenized L3 larvae, respectively. Infections with STm and Nb were administered intraperitoneally and subcutaneously respectively. Data is representative of 4–6 mice per group with experiments performed twice for each time point. (NS = Non-significant, ^*^P<0.05 and ^**^P<0.005).

STm and Nb induces immunoglobulin-switching to the Th1-reflecting IgG2a isotype or the Th2-reflecting isotypes IgG1 and IgE respectively. Furthermore, in this model of STm infection GC are absent early in the response, only becoming detectable later, when the infection has largely cleared [19]. As the direction of immunoglobulin-switching in mice can be influenced, in part by the cytokine milieu, it was possible that the altered cytokine environment during co-infection could alter the immunoglobulin-switching profile. In mice infected only with Nb, robust IgG1 and IgE EF plasma cell responses were detected, with IgG2a barely detectable by day 10 post-infection. This response was further characterised by an extensive GC response (Figure 5A). Mice infected with STm alone developed a robust IgG2a response with few IgG1 and no IgE cells detected. This response developed in the near total absence of GC, which only developed late in the response (Figure 5A). Surprisingly, in co-infected mice at days 10 and 32 post-infection a mixed switching-pattern was observed with IgG1, IgG2a and IgE plasma cells all readily detectable in EF foci. Interestingly, in co-infected mice development of the robust Nb-associated GCs was abrogated, only becoming detectable at day 32 post-infection (Figure 5A). Thus, the direction of B cell switching is maintained during co-infection, with the features of the response to each individual pathogen conserved.

### Co-infection diminishes serum immunoglobulin responses to STm

Antibody responses are dispensable for the control of primary STm infection in mice, although they play a central role in protecting against secondary infection and in vaccine responses [16], . Thus, we examined the impact of co-infection on serum antibody responses to both pathogens to identify if long-term protective immunity may be compromised by co-infection with Nb. Serum antibody responses against outer membrane antigens of STm at days 10, 18 and 32 post-infection were measured. Reflecting the early conserved EF plasma cell responses, IgM and IgG antibody titres were similar at days 10 and 18 post-infection in both groups that received STm (Figure 5B). In contrast, at day 32 post-infection when GC are detected, IgM, IgG and IgG2a titres were lower in co-infected mice relative to STm-only infected mice, despite the GC response being comparable between the two groups (Figure 5A). Measurement of specific antibody responses in Nb-only and co-infected mice revealed that serum anti-Nb IgM, IgG and IgG1 titres were reduced in co-infected mice relative to Nb-only infected animals at day 10 post-infection, when GC responses were diminished. However, by day 18 IgG responses were similar between the two groups as the GC start to become more established in co-infected animals (Figure 5A). Thus co-infection can impact serum antibody titres to each pathogen but does not necessarily alter the switching profile.

### Prior infection with Nb alters the control of STm and production of anti-STm IgG

**Figure 6:**
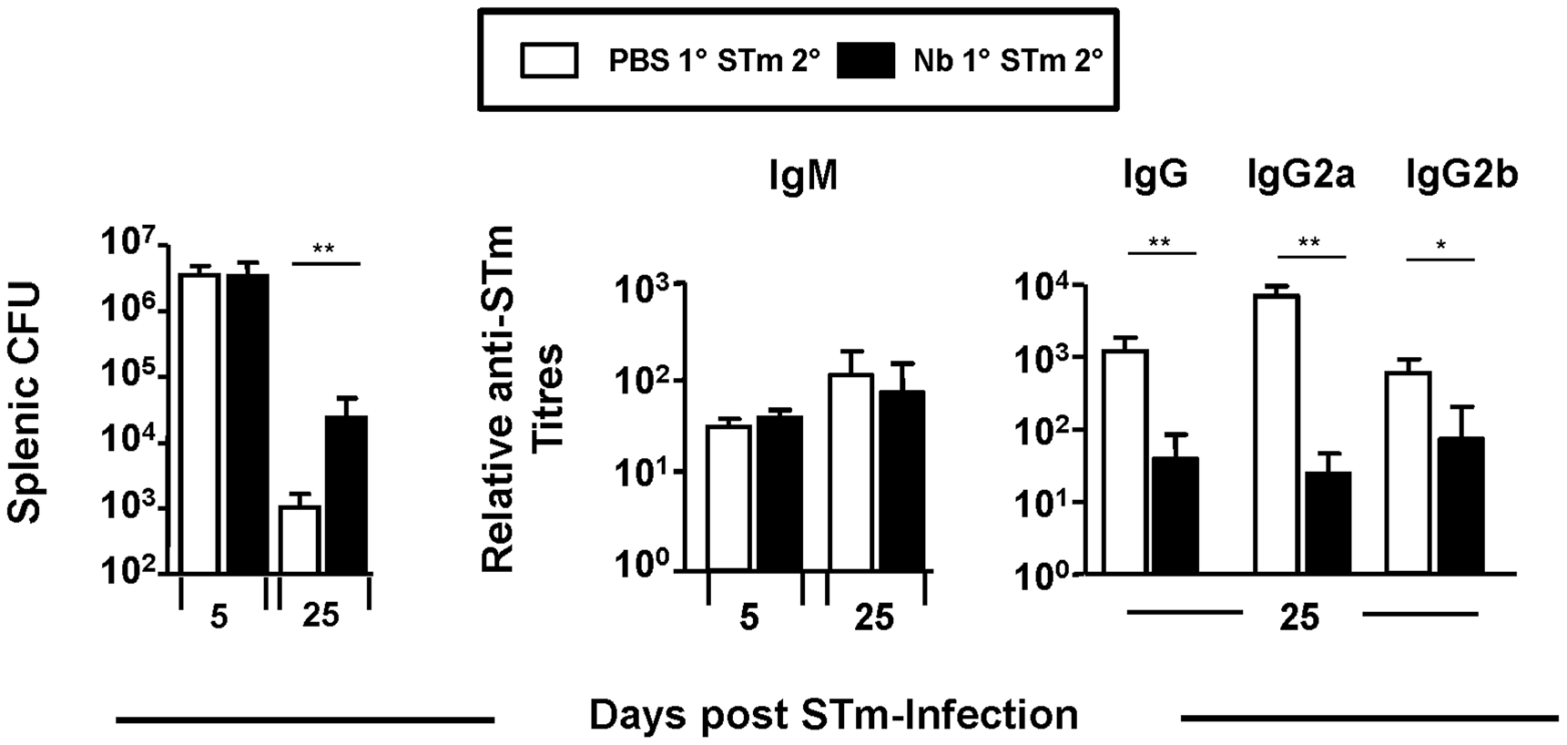
Prior Infection with Nb alters the control of STm and production of anti-STm IgG. WT mice were infected with 500 L3 Nb larvae and at day 16 mice were challenged with 5×10^5^ STm alongside naïve control mice. Splenic bacterial numbers were assessed at days 5 and 25 post-STm infection. Serum anti-STm IgM, IgG, IgG2a and IgG2b antibody titres were assessed by ELISA against a total outer membrane preparation of STm. Infections with STm and Nb were administered intraperitoneally and subcutaneously respectively. Groups contained 4–6 mice. (^*^P<0.05 and ^**^P<0.005).

Since simultaneous infection with Nb and STm could impair host control towards each pathogen the influence of sequential exposure was assessed. WT mice were infected with Nb and at day 16 (6–7 days post worm-expulsion) mice were challenged with STm for 5 or 25 days (Figure 6). While early control of STm was comparable between non-Nb primed and Nb-primed mice, prior Nb infection impaired control of STm at day 25, reflecting our earlier observations (Figure 1B). The impact of prior Nb infection on serum antibody responses to STm was then examined. This showed that antecedent Nb infection had no influence on anti-STm IgM titres but impaired anti-STm IgG titres at day 25 post-STm infection, with both IgG2a and IgG2b titres lower in mice previously infected with Nb (Figure 6). Thus prior Nb-infection can impair antibody switching to STm and the late control of subsequent STm infection.

### Prior Nb infection impairs the efficacy of immunization against STm with a subunit vaccine

**Figure 7:**
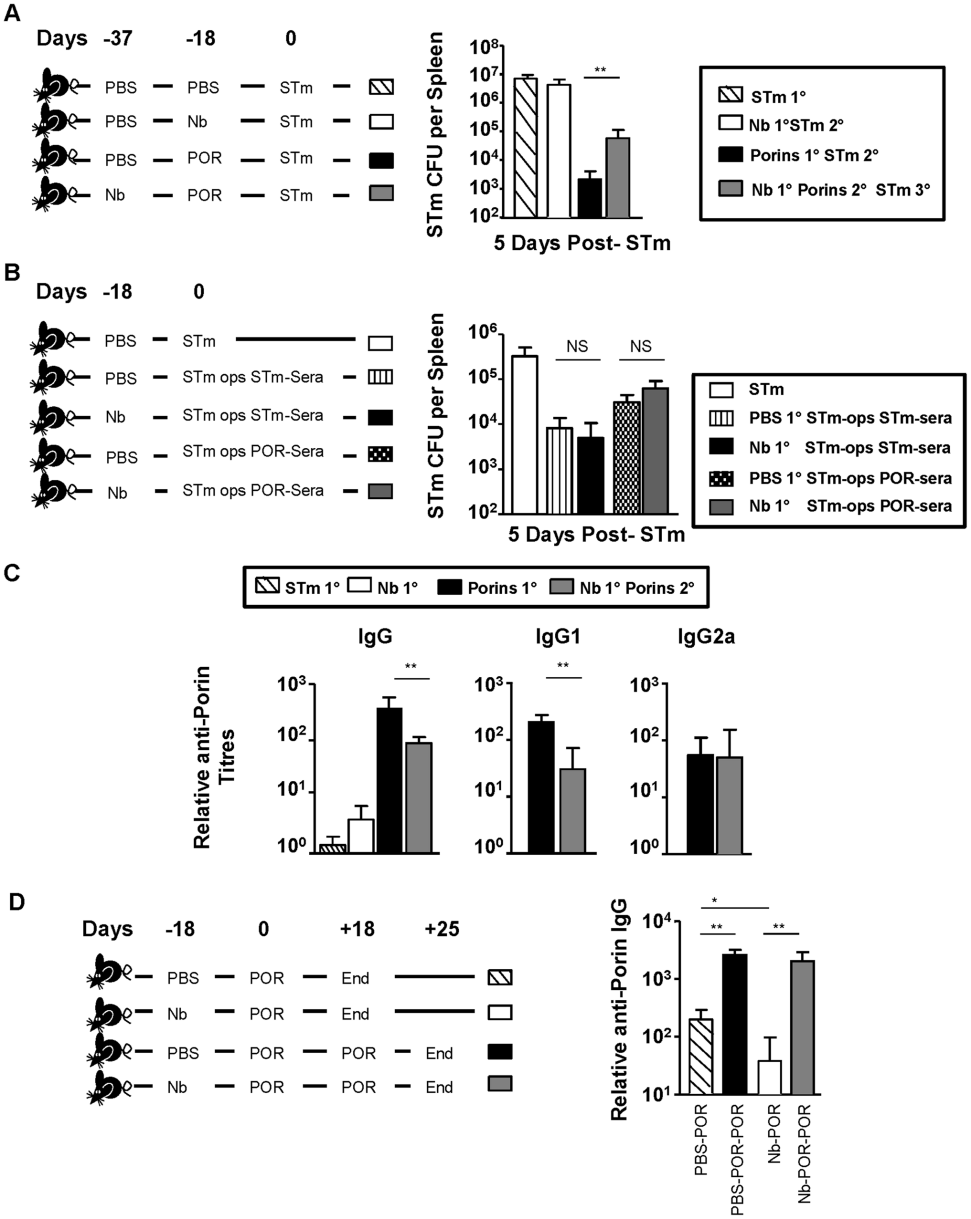
Prior Nb Infection impairs antibody titres and vaccine efficacy following porin-immunization. **A**) WT mice were infected with 5×10^5^ STm and splenic bacterial numbers were examined at day 5. Prior to infection mice were given either: i) PBS (dashed), ii) infected with 500 L3 Nb (open bar), iii) immunized with 20 µg porins (black bar) or iv) infected with 500 L3 Nb and then immunized with 20 µg porins (grey bar). **B**) WT mice were infected with 5×10^5^ STm opsonised with complement-inactivated serum from mice that had either been infected with STm for 35 days or primed with porins for 18 days and then boosted for 7 days. Splenic bacterial numbers were assessed 5 days post-infection. Prior to STm infection mice were either immunized with PBS or infected with 500 L3 Nb larvae for 16 days. Naïve control mice were infected with non-opsonised STm (open bar). **C**) Serum anti-porin IgG, IgG1 and IgG2a antibody titres were assessed by ELISA on serum isolated from mice immunized as in Figure 7A, but pre STm-infection. **D**) WT mice were either i) immunized with PBS (dashed bar) ii) infected with Nb for 18 days (open bar) before immunization with 20 µg porins for 18 days or iii) immunized with PBS (black bar) iv) infected with Nb for 18 days (grey bar) before immunization with 20 µg porins for 18 days followed by a second booster immunization for 7 days. Anti-porin IgG titres were then assessed by ELISA. Infections with STm and Nb were administered intraperitoneally and subcutaneously respectively. Data is representative of 4–6 mice per group and experiments were performed twice. POR = Porins. (^*^P<0.05).

Antibody induced during infection can protect against secondary STm infection [16], [19], [20], [22], [23]. Previously, we demonstrated that immunization with the porin proteins OmpC, D and F (collectively called porins) was sufficient to protect mice from STm infection via an antibody-dependent mechanism [26]. This offered an opportunity to examine the impact of prior Nb infection on antibody-mediated control of STm infection. To do this, groups of mice either received no intervention before STm infection, or combinations of Nb and porins before STm challenge (Figure 7A). After 5 days of infection splenic bacterial burdens were assessed. This showed that both porin-immunized groups had significantly lower bacterial numbers relative to non-immunized mice. Nevertheless, porin-immunized mice that had first been infected with Nb had a greater bacterial load than mice that had only received porins before infection (Figure 7A), indicating that Nb-infection can impair the protection conferred by porin-immunization.

### Infection with Nb reduces anti-porin titres but not the efficacy of antibody-mediated killing

Prior Nb infection may impact upon the success of immunization through at least two routes. Firstly, it may alter the activity of antibody, possibly through altering macrophage populations and their opsono-phagocytic capacity. Secondly, reduced benefit from immunization may reflect lower levels of antibody induction. To test the former, bacteria were opsonized with complement-inactivated sera from mice that had either been infected with STm or immunized with porins (Figure 7B). Opsonized bacteria were then given to mice i.p. that had either received PBS or Nb 18 days previously and bacterial burdens were enumerated 5 days later. In each case bacterial numbers recovered from mice infected with opsonized bacteria were similar irrespective of whether they had previously been infected with Nb (Figure 7B). This suggests there was no intrinsic defect in antibody-mediated control of STm infection in Nb-infected mice.

Since ≥95% of the protection provided by anti-porin antibody is through the induction of IgG [26], anti-porin antibody titres in mice immunized with porins after Nb infection were assessed. After immunization, porin-immunized Nb-infected mice had lower total anti-porin IgG serum titres than non-Nb infected counterparts (Figure 7C). Analysis of the distinct IgG isotypes induced showed there was diminution in IgG1 titres, whereas there was a negligible effect on IgG2a (Figure 7C). Therefore, prior Nb infection influences the titre of anti-porin IgG induced, but does not necessarily affect the efficacy of killing bacteria pre-opsonized with antibody. Finally, we looked to see if boosting with porins in Nb-infected mice could restore anti-porin antibody titres (Figure 7D). WT mice given PBS or Nb were immunized 18 days later with porins and 18 days after this some mice received a second porin-immunization. Antibody responses were then assessed after 7 days. Anti-porin IgG titres were similar in both boosted groups, irrespective of whether they were previously infected with Nb. This suggests that the reduced antibody titres observed after porin immunization can be restored through engagement of B cell memory.

## Discussion

This work identifies the mutual impairment in immune regulation when infection with Nb and STm occurs concurrently, as marked by the delayed clearance of STm and expulsion of Nb. This impaired host control was not limited to synchronous challenge with both pathogens as prior infection with Nb also impacted on the host response to STm and impaired vaccine-mediated protection, despite adult worms having been cleared. This indicates that the persistence of viable adult worms is not necessary for this effect, as described previously [42]. This is important as it supports the concept that the impact of infectious history or co-infection may not always require direct physical association between the pathogens, as shown with bacterial microflora and *Trichuris muris* [43].

The delay in STm clearance after infection with Nb was only apparent at times when adaptive immunity controls infection. Nevertheless, an impairment in the induction of Th1 cells or secretion of IFNγ after anti-CD3 stimulation was not obvious, nor was there a change in the levels of IFNγ after culture of splenocytes without stimulation. This suggests the underlying reason for defective immunity is not one of a failure to mount an appropriate immune response but may relate to other factors, such as the inefficient migration of T cells or inappropriate interactions between T cells and macrophages. Otherwise, the elevated IL-10 production observed in co-infected mice may alter the kinetics of STm clearance. Relevant to this perhaps is the increase in FoxP3 cells detected in the T zone after co-infection compared to STm alone. This may alter the functionality of T cells and limit their ability to promote bacterial clearance. Furthermore, during co-infection diminished, but not absent, Th2 cytokine secretion was observed and IL-4 and IL-13 were detectable after stimulation of splenocytes with killed STm. Although Th1 and Th2-associated responses can co-develop [44], in vivo and in vitro Th1 and Th2 cytokines have been shown to have opposing and suppressing activities [45], [46]. In the context of this study, only lower Th2 cytokine production was observed and this was partial, suggesting some potential Th1 dominance here, possibly because STm directly colonizes the spleen. Furthermore, IL-4 and IL-13 were both detectable in the day 32 STm-only group, probably reflecting the function of these molecules in GC development [47]. Nevertheless, it may be the balance between Th1 and Th2-associated cytokines, rather than the absolute amounts of each cytokine considered in isolation, which is the important factor. Such a consideration is relevant in other systems such as experimental *Leishmania major* infection [48]. Alternatively, this may simply reflect this specific combination of pathogens.

Other reasons may help account for the delayed control of STm infection. Levels of IL-4 and IL-13 were higher in non-stimulated splenocyte cultures from co-infected mice relative to mice only infected with STm. This may indicate other non-T cells contribute or impair clearance of STm through collaboration with T cells. Obvious candidates are innate lymphoid cells. Group 2 innate lymphoid cells (ILC2s) have been shown to release IL-13 in response to helminth infection [49] and recently the importance of ILC2s for the efficient development of Th2 cell responses during a Nb infection was demonstrated [38]. Therefore, in the same way that ILC2s can contribute positively to clearance of helminth infection they may impede the functioning of Th1 immunity. Many of the factors identified that potentially explain the failure to properly control STm infection in co-infected animals may also explain the delayed clearance of Nb. The cytokine most associated with efficient clearance of helminth infection is IL-13. Therefore, the diminished IL-13 cytokine production detected, in combination with the elevated levels of IFNγ, may inhibit the rate of worm expulsion. Other reasons that could help account for the delayed clearance of Nb include reduced levels of IL-4 production or a reduced expression of the respective receptors for IL-4 and IL-13 on cells such as smooth muscle cells [11], [50] or B cells [14]. The intermediate levels of FoxP3 T cells observed during co-infection may paradoxically have a negative effect on Nb clearance through enhancing Th1 inflammation and thus restricting the limited Th2 response induced from functioning.

Furthermore, in responses to other helminths loss of MyD88 in mice can enhance protection [51]. Therefore it may be that strong engagement of this molecule, for instance through the multiple TLRs triggered by STm, inhibits immunity. These factors could collaborate to limit the efficacy of the Th2 response induced and diminish the efficiency of worm clearance. One possibility to consider is if the addition of exogenous Th2 cytokines would recapitulate the protective immunity to Nb seen in the absence of STm co-infection. We would expect not for two reasons. First, the presence of Nb during STm infection has virtually no impact on IFNγ production, suggesting that the pro-inflammatory cytokine profile and possibly its anti-Th2 activities would be retained. Second, relates to the technical complexity of delivering IL-4 or IL-13 sufficient within the host to overcome this inhibition. This can be achieved by delivering these cytokines through a pump or as a complex with antibodies [52], although being able to provide this continuously and throughout infection would be challenging and prohibitive.

Antibody plays an important role in preventing re-infection with STm and the appearance of antibody to the pathogen correlates with reduced risk of bacteraemia in infants, but in the mouse it is not required for the control of primary infection [53]. Furthermore, the Vi capsular polysaccharide vaccine against typhoid works via the induction of antibody [54] and provides equivalent protection in the first few years after administration as the live, attenuated vaccine. Thus understanding how optimal levels of antibody to STm are induced is important to understand the mechanisms of control to this pathogen. STm alone failed to induce GC in the first weeks of infection, whereas Nb-infection induced pronounced GC responses and co-infection resulted in the abrogation of this response to Nb. Therefore, whilst the direction of EF switching in the spleen is largely independent of the presence of a second pathogen, the development of GC responses is not. In vitro and in vivo IL-4 is essential for directing B cell switching to IgE [55], but is dispensable for IgG1 switching [56]. Unexpectedly, EF IgG1 and IgE switching in the spleen was detectable at similar levels in both co-infected and Nb-only infected mice, despite reduced levels of IL-4 after co-infection. This implies that whilst IL-4 is essential for IgE switching, it may only be required at low levels. Furthermore, the augmented levels of Th2 cytokines during co-infection did not moderate the induction of IgG2a to STm. Therefore, both Th1 and Th2 cell priming and the characteristic class-switching profile is conserved and co-developed in the same responding secondary lymphoid tissue during co-infection. This is compatible with our earlier observations immunizing with soluble flagellin and flagellated bacteria where the direction of antibody-switching was conserved relative to the direction of T cell differentiation [15], [57]. This is important as it indicates that only selective elements of immunity are influenced by the presence of infecting organisms.

Despite EF switched plasma cell numbers being similar between co-infected mice and mice challenged with either STm or Nb there were some effects of co-infection on antibody titres. The anti-STm antibody response was similar between both STm-infected groups at day 18, yet at day 32, a time when antibody would largely originate from the GC, there was a clear reduction in IgM and switched antibody titres despite no difference in the splenic area occupied by GC. One possibility is that although the total number of GC may be similar between STm-only and co-infected mice at day 32, some of the GC in co-infected mice are Nb-specific and others STm-specific. Alternatively, it may relate to the higher bacterial burdens seen on day 32 in co-infected mice, which can alter the kinetics of GC induction [19] or other factors may be involved. Such influences may also explain why there was a lasting influence of Nb infection on anti-STm IgG antibody titres when Nb infection preceded STm infection. This impact on antibody titres was not restricted to live STm as the antibody response to STm porins was also lower when administered after Nb infection. Lower IgG titres were associated with diminished protection from infection, whilst the capacity of Nb-infected mice to control infection with antibody-opsonized STm was similar to non-Nb infected controls. This suggests that the capacity of cells to phagocytose and kill STm is not influenced by Nb-infection since antibody does not kill STm via cell-free complement-mediated mechanisms in mice [58]. Anti-NTS IgG strongly correlates with lower risk of invasive NTS infection in humans [53], and our study implies that the level of anti-porin IgG titres may influence protection. Whether co-infection with STm and helminths in humans is associated with altered IgG titres to STm and risk of infection needs to be addressed.

Helminth infections in humans are associated with lower vaccine efficacy to subunit and live vaccines [30]–[33], [59]. For instance, helminth infections are associated with diminished IgG and IgA antibody responses to cholera toxin B subunit [60] and to a live-attenuated oral cholera vaccine strain [61]. Interestingly, while treatment for helminth infection prior to vaccination can improve vaccine responses [61] our results indicate that prior infection could continue to have a detrimental effect on efficacy, although this that can be circumvented by antigen boosting.

In summary, helminth infections can influence antibody responses to STm and subunit vaccines and this should be considered when translating findings generated in animal models into humans, particularly in regions endemic for helminths. Understanding how helminths influence antibody induction will help us identify how best to employ vital life-saving vaccines. As antibody titres to porins post-Nb infection reached normal levels after boosting it would suggest that exploiting memory B cell responses would be important for the efficacy of subunit vaccines in helminth-endemic regions.
